# Whole-Genome Sequences of One Arthrobacter Strain and Three Pseudarthrobacter Strains Isolated from the Namib Desert

**DOI:** 10.1128/MRA.00885-19

**Published:** 2019-10-24

**Authors:** Elizabeth Buckley, Kevin C. Lee, Colleen M. Higgins, Brent Seale

**Affiliations:** a School of Science, Faculty of Health and Environmental Sciences, Auckland University of Technology, Auckland, New Zealand; University of Arizona

## Abstract

We report here the draft genome sequences of one Arthrobacter sp. strain (NamB2) and three Pseudarthrobacter sp. strains (NamE2, NamB4, and NamE5) isolated from topsoil in the Namib Desert, Namibia. The assemblies contain between 29 (NamB2) and 68 (NamE5) contigs >200 bp and range from 3.26 Mb (NamB2) to 4.03 Mb (NamE2). One plasmid was identified in *Pseudarthrobacter* sp. strain NamE5.

## ANNOUNCEMENT

One Arthrobacter sp. strain (NamB2) and three Pseudarthrobacter sp. strains (NamE2, NamB4, and NamE5) were isolated from open soil in the Namib Desert, Namibia, following collection in April 2017. The coordinates of the sample sites are 23°01.016′S, 014°51.547′E (isolates NamB4 and NamE2), 23°08.554′S, 015°12.578′E (isolate NamE5), and 23°14.714′S, 015°21.663′E (isolate NamB2). The soil was diluted in phosphate-buffered peptone (BD Difco, USA) at a ratio of 1 g to 10 mL, and a 100-μl suspension was inoculated onto nutrient agar (NA; BD Difco) and Reasoner's 2A (R2A) agar (Neogen, UK), before aerobic incubation at 15 to 20°C for 1 week. Strains NamB2 and NamE2 were isolated on NA, and NamB4 and NamE5 were isolated on R2A agar. Single bacterial colonies were subcultured and identified via screening and selection for survival under different laboratory conditions. Isolates were grown in Difco nutrient broth (BD) from single colonies and stored in 25% (vol/vol) glycerol stocks at –80°C until required.

For Sanger sequencing of the 16S rRNA gene, strains were grown in nutrient broth for 4 days from 25% (vol/vol) glycerol stocks of the original subculture. *Arthrobacter* sp. strain NamB2 shared 99.63% 16S rRNA gene sequence identity with A. agilis DSM 20550^T^ (GenBank accession number NR_026198), using universal 27F-1492R primers for amplification, Sanger sequencing (Macrogen, South Korea), and BLASTn for comparison (https://www.nlm.nih.gov/). *Pseudarthrobacter* sp. strains NamB4, NamE5, and NamE2 showed, respectively, 99.33%, 99.48%, and 98.04% 16S rRNA gene sequence identity with Pseudarthrobacter phenanthrenivorans Sphe3^T^ (GenBank accession number NR_074770), using the same approach as that for NamB2.

Genomic DNA was extracted using a modified cetyltrimethylammonium bromide (CTAB) method, as previously described (1). The genome of each strain was sequenced by Illumina sequencing (HiSeq SBS kit v4 chemistry at 2 × 125 bp) on an Illumina HiSeq 2500 instrument by Genewiz (Suzhou, China). The number of raw reads generated for each strain were 51,352,518 for NamB2, 56,223,629 for NamE2, 34,774,522 for NamB4, and 35,472,273 for NamE5. Default parameters were used for all software unless otherwise specified. The coverage of the reads was first normalized based on kmer frequency with BBnorm (https://sourceforge.net/projects/bbmap/) to produce reads with an average depth of 100× and by discarding reads with an apparent depth below 5× to reduce the effect of irregular coverage on the assembler. The normalized reads were then assembled using the Unicycler pipeline v3.12.0 (2), which calls and optimizes assembly by SPAdes v 3.12.0 (3). Table 1 shows the features of each draft genome with the number of contigs generated for the genome of each organism, the G+C content, and the estimated genome size. Following the genome assembly, it was found that *Pseudarthrobacter* sp. NamE5 contained a plasmid, pE5100, represented by a single contig of 227,930 bp and a G+C content of 60.2%. While an origin of replication sequence was not detected on the plasmid, we believe this to be a plasmid due to the De Bruijn assembly graph showing a single contig in a circular path and due to the size of the plasmid being a typical size for *Actinobacteria* (4).

**TABLE 1:** Features of draft genomes for the sequenced Arthrobacter strain NamB2, Pseudarthrobacter strains NamE2, NamB4, and NamE5, and the plasmid pE5100, as annotated by NCBI PGAP^*a*^

Isolate	Genome coverage (×)	*N*_50_ value (bp)	Genome size (bp)	G+C content (%)	No. of contigs >500 bp	No. of protein coding genes	No. of rRNA genes (5S, 16S, 23S rRNA)	No. of tRNA genes
NamB2	137	487,726	3,260,467	67.6	22	2,897	3 (1, 1, 1)	46
NamE2	136	235,942	4,035,682	65.3	52	3,609	3 (1, 1, 1)	50
NamB4	134	200,647	3,818,763	64.9	62	3,354	3 (1, 1, 1)	50
NamE5	142	203,936	3,830,394	64.6	42	3,634	3 (1, 1, 1)	50
pE5100 plasmid	142	227,930	227,930	60.2	1	235	0	0

a PGAP, Prokaryotic Genome Annotation Pipeline.

Gene prediction and annotation were carried out using the NCBI Prokaryotic Genome Annotation Pipeline (PGAP) (5). PGAP annotation predicted 2,897 protein-encoding genes for *Arthrobacter* sp. NamB2, 3,614 for *Pseudarthrobacter* sp. NamE2, 3,354 for *Pseudarthrobacter* sp. NamB4, and 3,609 for *Pseudarthrobacter* sp. NamE5. The pE5100 plasmid appeared to have 235 protein coding genes. The number of predicted tRNA genes for each isolate was variable; there were 46 for NamB2 and 50 each for NamE2, NamB4, and NamE5. No CRISPR regions were found in the Namib isolates using MinCED (6) software.

### Data availability.

The draft genome sequences of the Namib isolates were deposited at the DDBJ/ENA/GenBank under the accession numbers SZWI00000000, VAHM00000000, VAHN00000000, and VAHO00000000. The pE5100 plasmid was deposited at the DDBJ/ENA/GenBank under the accession number CM016730.
